# Predicting the Internal Knee Abduction Impulse During Walking Using Deep Learning

**DOI:** 10.3389/fbioe.2022.877347

**Published:** 2022-05-12

**Authors:** Issam Boukhennoufa, Zainab Altai, Xiaojun Zhai, Victor Utti, Klaus D McDonald-Maier, Bernard X. W. Liew

**Affiliations:** ^1^ School of Computer Science and Electrical Engineering, University of Essex, Colchester, United Kingdom; ^2^ School of Sport, Rehabilitation and Exercise Sciences, University of Essex, Colchester, United Kingdom

**Keywords:** gait biomechanics, knee joint moments, machine learning, neural network, time-series

## Abstract

Knee joint moments are commonly calculated to provide an indirect measure of knee joint loads. A shortcoming of inverse dynamics approaches is that the process of collecting and processing human motion data can be time-consuming. This study aimed to benchmark five different deep learning methods in using walking segment kinematics for predicting internal knee abduction impulse during walking. Three-dimensional kinematic and kinetic data used for the present analyses came from a publicly available dataset on walking (participants *n* = 33). The outcome for prediction was the internal knee abduction impulse over the stance phase. Three-dimensional (3D) angular and linear displacement, velocity, and acceleration of the seven lower body segment’s center of mass (COM), relative to a fixed global coordinate system were derived and formed the predictor space (126 time-series predictors). The total number of observations in the dataset was 6,737. The datasets were split into training (75%, *n* = 5,052) and testing (25%, *n* = 1685) datasets. Five deep learning models were benchmarked against inverse dynamics in quantifying knee abduction impulse. A baseline 2D convolutional network model achieved a mean absolute percentage error (MAPE) of 10.80%. Transfer learning with InceptionTime was the best performing model, achieving the best MAPE of 8.28%. Encoding the time-series as images then using a 2D convolutional model performed worse than the baseline model with a MAPE of 16.17%. Time-series based deep learning models were superior to an image-based method when predicting knee abduction moment impulse during walking. Future studies looking to develop wearable technologies will benefit from knowing the optimal network architecture, and the benefit of transfer learning for predicting joint moments.

## 1 Introduction

Knee joint moments are commonly calculated to provide an indirect measure of knee joint loads (Webster et al., 2012; Richards et al., 2018a; Robinson et al., 2021), with the frontal plane moment being the most implicated as a risk factor for the onset, exacerbation, and relapse of knee pathologies. For example, in knee osteoarthritis (OA), a greater external knee adduction moment (KAM) has been linked with accelerated disease progression (Henriksen et al., 2014). In contrast, a greater external knee abduction moment has been linked with a greater risk of developing patellofemoral pain and anterior cruciate ligament (ACL) injuries (Myer et al., 2015). Researchers have begun coupling real-time biofeedback of the KAM, together with provision of specific gait modification strategies in people with knee OA during walking, and this resulted in up to 14% reduction in peak KAM (Richards et al., 2018b).

Inverse dynamics represent the “Gold Standard” to calculate knee joint moments (Ren et al., 2008; Holder et al., 2020; Baltzopoulos, 2021). A shortcoming of inverse dynamics approaches is that the process of collecting and processing human motion data can be time-consuming (Ren et al., 2008; Holder et al., 2020; Baltzopoulos, 2021). The traditional methods to calculate knee joint moments makes it clinically impractical for large scale implementation. To circumvent the need to use inverse dynamics, researchers have turned to machine learning to predict knee joint moments (Favre et al., 2012; Aljaaf et al., 2016; He Z. et al., 2019; Mundt et al., 2020a; Mundt et al., 2020b; Stetter et al., 2020; Wang et al., 2020; Boswell et al., 2021)—which we term, the ML approach.

The most common ML method used to predict joint moments is neural networks (He Z. et al., 2019; Stetter et al., 2020; Wang et al., 2020; Boswell et al., 2021). Some studies adopt a shallow network architecture with one/two “hidden” layers (Favre et al., 2012; He Z. et al., 2019; Stetter et al., 2020), whilst others used deeper layers (Wang et al., 2020; Boswell et al., 2021). Studies have used kinematics captured using markerless motion capture (Boswell et al., 2021), a combination of inertial measurement units (IMUs) and plantar pressure insoles (He Z. et al., 2019), and IMUs alone (Stetter et al., 2020; Wang et al., 2020) as predictors. Currently, body kinematics obtained *via* IMUs represent the best variables used for predicting joint moments, compared to variables like plantar pressure from insoles and ground reaction forces from force plates. This is because IMUs, in contrast to pressure insoles, are commercially ubiquitous and cost-effective, and are the most accurate commercial devices to capture kinematics when compared to three-dimensional motion capture (Slade et al., 2022). Also, IMUs in contrast with force plates can be used in free-living environments. Outcomes that have been predicted include the peak value (He Z. et al., 2019; Boswell et al., 2021), the waveform (Stetter et al., 2020; Wang et al., 2020), and the moment integral (Stetter et al., 2020) of KAM. Reported prediction accuracies for KAM during walking range 0.03 Nm/kg to 0.15 Nm/kg (Table 1).

**TABLE 1:** Non-exhaustive list of studies using macine learning to predicting knee moments.

Study	Predictors	Outcome	Machine-learning algorithm	Performance error	Performance error—scaled*
Boswell et al. (2021)	3D positions of 13 anatomical landmarks	Peak external knee adduction moment	Fully connected neural network (10 hidden layers)	MAE: 0.53% (BW.Ht)	0.15 Nm/kg
Favre et al. (2012)	12 kinematic and kinetic variables	External knee adduction moment waveform	Multi-layer perceptron neural network (1 hidden layer)	MAD: 0.36% (Bw.Ht)	0.06 Nm/kg
Wang et al. (2020)	4 demographic, 24 kinematic variables	External knee adduction moment waveform	Fully connected neural network (10 hidden layers)	MAE: 0.002 Nm/(BW.Ht)	0.03 Nm/kg
He et al. (2019b)	24 plantar pressure variables	External knee adduction moment waveform	Multi-layer perceptron neural network (1 hidden layer)	RMSE: 0.36% (BW.Ht)	0.06 Nm/kg
Stetter et al. (2020)	16 kinematic variables	Peak external knee adduction moment	Fully connected neural network (2 hidden layer)	Difference: 0.11 Nm/kg	0.11 Nm/kg

*Scaled based on 1.70 m tall participant.

Abbreviations: MAE, mean absolute error; MAD, mean absolute deviation; RMSE, root mean squared error

Most studies that used neural networks contain fully connected layers as their model architecture (Favre et al., 2012; He Z. et al., 2019; Stetter et al., 2020; Wang et al., 2020; Boswell et al., 2021). A limitation of fully connected layered neural networks is that more parameters have to be learned, thereby increasing computation time, compared to convolutional networks. Another limitation of fully connected layered neural networks in biomechanics is that they cannot accommodate temporal variables. To circumvent this limitation, researchers have opted to treat each value of a time-series as independent observations (Stetter et al., 2020), which ignores the inherent correlation in temporal biomechanics data.

Boswell et al. (2021) trialed different time-series neural network architectures (e.g., long short-term memory [LSTM]) but reported these to be inferior to a fully connected network. The findings of Boswell et al. (2021) was surprising given that LTSM are examples of time-series models that takes into account the information (e.g., correlation) from adjacent time-points to model the relationship between the predictors and outcome. The lack of benefit of using a LTSM model over a fully connected network could be explained by the relatively shallow number of layers (2 LTSM layers) (Boswell et al., 2021), which may have precluded learning an adequate representation of the prediction problem. However, a well-known problem of having deep layers in a neural network is the “vanishing gradient” issue (Veit et al., 2016), which states at deeper layers, there is incrementally lesser amount of information available for learning new relationships.

In this work, we employed more sophisticated time-series models consisting of the InceptionTime (Ismail Fawaz et al., 2020) and TS-ResNet (Wang et al., 2017), which till this current work to the authors knowledge, have not been used in biomechanics. These network architectures have been inspired by the popular Inception-v4 and the Resnet architecture that performed outstandingly in the computer vision domain. InceptionTime combines five deep learning models, each consisting of multiple Inception blocks (Ismail Fawaz et al., 2020). The inceptionTime outperformed other neural network architectures (convolutional neural networks [CNN], LSTM, bidirectional [BiLSTM], CNN-LSTM, and Gated Recurrent Units [GRU]) in human activity recognition (Pantawong et al., 2021). TS-ResNet enables very deep layers by adding connections that skip over some layers, to slow the rate of learning reaching saturation, and thus avoid the degradation problem. TS-ResNet outperformed eight neural network architectures on 97 time-series datasets (Ismail Fawaz et al., 2019). Both these networks have not been used in joint moment estimation which is therefore a contribution of this work. In addition, time-series data can also first be encoded into images using Gramian Angular Field (GMF) and then fed into neural network architectures typically used for image-based datasets—a technique that significantly improved human activity recognition compared to traditional CNN models (Boukhennoufa et al., 2021a).

A challenge when using ML in biomechanics is the sample size. When the sample size is small, the ML model may generalize poorly for new observations. Current studies have used data from a very small cohort of 10 (Liu et al., 2009) to a larger cohort of 106 participants (Wang et al., 2020). However, even though a sample size of 100 participants is considered large clinically, it pales in comparison to non-clinical ML research [e.g., millions of samples (Simonyan and Zisserman, 2014)]. A novel ML method to manage the issue of small sample sizes is transfer learning (Weiss et al., 2016). Transfer learning takes advantage of “knowledge” from existing large pre-trained ML models, with the collected biomechanical data used for fine-tuning (Johnson et al., 2019). Pre-trained models such as the VGG network, have been trained on 1.3 million ImageNet images and 1000 object classes (Simonyan and Zisserman, 2014).

Transfer learning achieved a Pearson correlation of 0.94–0.97 (Johnson et al., 2019), which was similar to the correlation of 0.96 when using a custom CNN model (Wang et al., 2020) for predicting knee abduction/adduction moments during walking. Interestingly, a direct benchmark study in running reported that transfer learning resulted in poorer correlation compared to a custom CNN model (Liew et al., 2021). However, the previous study converted the time-series to images for modelling which could have introduced “noise” to the original data (Liew et al., 2021). To our knowledge, no studies have benchmarked the performance of different state-of-art time-series and image-based network architectures, and transfer learning for predicting knee joint moments in walking.

The potential clinical benefit of being able to quantify knee joint moments in the field warrants a systematic investigation of the optimal network architectures that maximizes prediction performance. Given that a previous study has reported that moment integrals (i.e., impulse) provide a better indication of joint load than peak moment values (Kean et al., 2012), this study aimed to benchmark different neural network architectures in using walking segment kinematics in predicting internal knee abduction impulse during walking. The decision to report internal and not external moments will be explained in the Methods section. We hypothesized that transfer learning will perform better than a simple custom CNN model in predicting internal knee abduction impulse.

## 2 Materials and Methods

### 2.1 Design

This was a secondary analysis of a publicly available dataset, using a single session, cross-sectional laboratory study design (Fukuchi et al., 2018). Hence, no ethical approval was required for the conductance of this secondary analysis.

### 2.2 Dataset

The data came from a public dataset of 42 healthy adults walking on a treadmill, the details of which can be found in the original open-source publication (Fukuchi et al., 2018). Nine out of the 42 participants from the walking dataset were excluded from the present study. These participants had simultaneous bilateral foot contacts on the same force plate, resulting in an absence of consecutive good foot contact strides which lasted >50% of the walking duration. The 50% threshold was determined by the authors to minimize manual identification of foot contact events, to increase processing replicability (Liew et al., 2019).

Participants performed unshod walking on a dual-belt, force-instrumented treadmill (300 Hz, FIT; Bertec, Columbus, OH, United States), and motion was captured with 12 opto-electronic cameras (150 Hz, Raptor-4; Motion Analysis Corporation, Santa Rosa, CA, United States) (Fukuchi et al., 2018). This dataset was deemed feasible for this study given that the primary aim is to determine the optimal network architecture for using time-series kinematic measures to predict knee joint moment impulse. Walking occurred over eight controlled speeds: 40%, 55%, 70%, 85%, 100%, 115%, 130%, and 145% of each participant’s self-determined dimensionless speed (Froude number). The associated absolute walking speeds for all eight conditions for each participant were reported by the authors (Fukuchi et al., 2018). Marker trajectories and ground reaction force (GRF) were low passed filtered at a matched frequency of 6 Hz (4th Order, zero-lag, Butterworth) (Liew et al., 2019). A seven-segment lower limb, 6DOF joint model was developed in Visual 3D software (C-motion Inc., Germantown, MD, United States) (Liew et al., 2019). A force plate threshold of 50N was used to determine gait events of initial contact and toe-off.

Three-dimensional (3D) angular and linear displacement, velocity, and acceleration of the seven segment’s center of mass (COM), relative to a fixed global coordinate system were derived and formed the predictor space (126 time-series predictors).These kinematic predictors were used as it represented predictors can potentially be measured using IMUs. Internal moments are automatically calculated in Visual 3D. Hence, the internal knee abduction moment (inverse of the external KAM) was calculated using inverse-dynamics and expressed in the proximal segment’s reference frame (Schache and Baker, 2007) (negative values indicated internal knee abduction moment).

### 2.3 Machine Learning Modeling

All analyses were done using Python (version 3.7.0), with packages (Numpy v1.19.5, Pandas v1.1.5, Scipy v1.4.1). All ML models were trained using either Keras (version 2.4.0) or Tsai (version 0.2.2) from fastai with Google Collab’s Tesla V100 GPU, 25 GB RAM.

### 2.4 Generic Pre-processing

All time-series (predictors and outcome) were segmented between initial contact and toe-off (Liew et al., 2019). For the outcome, the area under the (negative) internal knee abduction moment curve for each time-series segment was calculated to provide a measure of knee abduction impulse. The knee abduction impulse was normalized to each participant’s body mass (Nm.s/kg). Given that the stance duration between each step, each speed condition, and participants were different, each time-series segment had a different number of data points. We zero-padded the time-series segments to have an equal number of data points as that of the longest time-series segment (Dwarampudi and Reddy, 2019; Pogson et al., 2020).

We explored three different pre-processing methods and their influence on prediction performance: 1) using raw time-series data as predictors, 2) normalizing the time-series predictors to a range from 0 to 1, and 3) standardizing the time-series predictors to a mean of 0 and standard deviation of 1. Although scaling of predictors (e.g., to a mean of 0 and standard deviation 1) is commonly advocated in ML (Burdack et al., 2020), our exploratory analysis revealed that using raw time-series as predictors provided the best prediction performance, and this was subsequently used in formal ML modelling.

The total number of observations in the dataset was 6,737 corresponding to 6,737 participant-steps. The predictor dataset was organised into a 3D array of shape 6737×126 ×300 , where the second dimension was the number of predictors, and the third dimension was the number of time points. The outcome dataset was organised into a 1D vector of length 6737 . Both the predictor and outcome datasets were split into training (75%, *n* = 5,052) and testing (25%, *n* = 1685) datasets (Wouda et al., 2018). The training dataset contains 75% of all the participants’ data with all the controlled speeds while the test dataset contains the rest of the dataset over the controlled speeds. This allows the model to learn from all the different cases to permit a more robust generalisation for each distinct instance. Our method of ML model development relies on a scenario that a participant comes for a baseline biomechanics assessment to develop a personalised model for the prediction of future instances of knee joint loads.

### 2.5 Algorithms

The following architectures were evaluated: 1) A 2D CNN-based model used as a baseline model, 2) InceptionTime model, 3) transfer learning, 4) the TS-Resnet model, and 5) the combination GADF-xResnet18. We have specifically selected the TS-Resnet, the InceptionTime and the combination GADF-xResnet18 as they have been very successful in dealing with other TS domains such as in activity recognition (Boukhennoufa et al., 2021a; Boukhennoufa et al., 2021b) and we wanted to investigate the performance in predicting the knee abduction moment impulse.

#### 2.5.1 2D CNN Model

**FIGURE 1:**
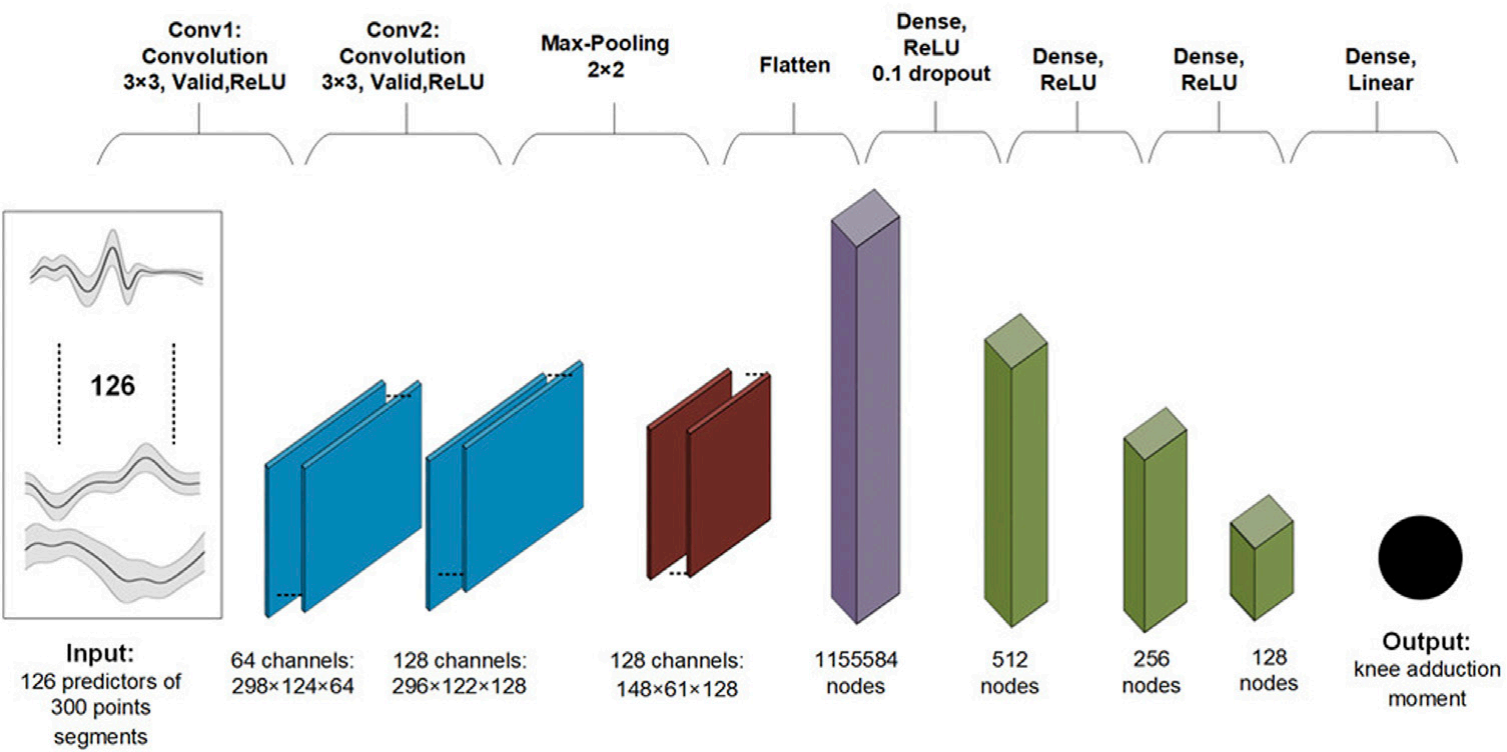
Baseline two dimensional convolutional neural network architecture.

The baseline 2D CNN model architecture can be found in Figure 1. Convolutional layers in a neural network are designed to learn a hierarchical representation of local features (e.g., peaks) of the predictors (Indolia et al., 2018). Advantages of convolutional layers over fully connected layers include having to learn much fewer parameters, better generalizability, and better scalability to big datasets. The model hyperparameters were selected based on initial exploratory analysis. Neural network (NN) weights were initialized with Xavier initialization (Glorot and Bengio, 2010). The Xavier initialization method is calculated as a random number with a uniform probability distribution (U) between the range $-\;\frac{1}{\sqrt{n}}$ and $\frac{1}{\sqrt{n}}$ , where n is the number of inputs to the node.

$$weight=U\left[-\;\frac{1}{\sqrt{n}}\;,\frac{1}{\sqrt{n}}\;\right]$$

A batch size of 64, 100 epochs of training repetitions, a learning rate of 3e^−3^, and an Adam optimiser were used. We used the mean squared error as the loss criteria.

For the other neural network models, a different method to find the appropriate learning rate, which has been termed cyclical learning rates, was used (Smith, 2017). The loss was plotted with respect to an increasing value of the learning rate. The learning rate was chosen to be in the interval that resulted in the lowest loss, which was found to be between 8e^−3^ and 1e^−1^. The learning rate took the value of 8e^−3^ at the first epoch and then gradually increased to reach a final value of 1e^−1^ at the last epoch. In conjunction with the cyclical method, it was found that after only ten epochs the loss stabilises and therefore 10 epochs were chosen, and a batch size of 128. weights were initialized with Xavier initialisation. The three models used are described below:

#### 2.5.2 InceptionTime

InceptionTime consists of an ensemble of deep CNN models, inspired by the Inception-v4 architecture for computer vision (Ismail Fawaz et al., 2020). The composition of an Inception network contains two different residual blocks. For the Inception network, each block is comprised of three Inception modules rather than traditional fully convolutional layers. Each residual block’s input is transferred *via* a shortcut linear connection to be added to the next block’s input. Following these residual blocks, we employed a Global Average Pooling layer that averages the output multivariate time-series over the whole time dimension. Each inception module contains a bottleneck 1D CNN layer with 32 output channels, a stride of 1 and a kernel size of 1 to reduce parameter dimensionality. The bottleneck layer is followed by three 1D CNN layers with an output channel of 32, a kernel size of 39, 19, 9 consecutively, a padding of 19, 9 and 4, with a stride of 1 in all the cases. The final layer of the InceptionTime network consists of a linear layer to output the internal knee abduction moment impulse.

#### 2.5.3 Transfer Learning InceptionTime

The InceptionTime model previously defined that was pre-trained on datasets from the UCR archive (Dau et al., 2019). Only two layers were tuned from the InceptionTime model - the first input layer to ensure that the required dimensions of the data conformed to our dataset; and the last layer in which the activation function was changed to linear to predict the continuous outcome of knee abduction moment impulse.

#### 2.5.4 TS-Resnet

ResNet allows using very deep structures which minimises the problems of vanishing gradients and accuracy saturation, by adding shortcut connections in each residual block to enable the gradient flow directly through the bottom layers (He et al., 2016). A residual block is a stack of layers set in such a way that the output of a layer is taken and added to another layer deeper in the block. The non-linearity is then applied after adding it together with the output of the corresponding layers in the main path. A time-series residual block is comprised of stacking three 1D CNN layers followed by a batch normalization layer and a ReLU activation layer. The number of filters for the CNN layers in each residual block are 64 then 128 then 256. The final ResNet stacks three residual blocks followed by a global average pooling layer and finally a linear activation layer to predict the knee abduction moment impulse.

#### 2.5.5 GADF-xResnet18

This model required the 1D time-series predictors to be converted into 2D images. To do so, we used the Gramian Angular Difference Fields (GADF) algorithm (Wang and Oates, 2015), which takes as an input the 6737×126 ×300 time-series sequences and outputs 6737×126 images of sizes 300 ×300 i.e: 6,737 images for each of the 126 predictors. GADF is a time-series encoding method that represents each time-series into 2D polar coordinates presented in a matrix-form called the Gramian matrix. Each element of this matrix is the difference (GADF) of their sine values. This mapping maintains the temporal dependency of the time-series. This was undertaken for both the training and testing predictor datasets.

The images encoded using GADF was than fed into the xResNet18 model (He T. et al., 2019) which is an improvement of the conventional ResNet18 that consists of 1) moving the stride 2 from the first convolutional layer to the second convolutional layer in the residual block, 2) removing the 7 × 7 convolution in the input layer of the network and replacing it with three consecutive 3 × 3 convolutions, and finally 3) adding a 1 × 1 convolution of stride 2 at the end of the residual block to reduce the number of parameters.

#### 2.5.6 Predictive Performance

Prediction performance of the knee abduction impulse was calculated on our test dataset using the metrics of the root mean squared error (RMSE, Nm.s/kg), the mean average error (MAE, Nm.s/kg), and the mean absolute percentage error (MAPE, %), and the normalized root mean squared Error (NRMSE, %) (Gholami et al., 2020). The RMSE was computed using

$$RMSE=\sqrt{\frac{\sum_{i=1}^{N}\|y\left(i\right)-\hat{y}\left(i\right){\|}^{2}}{N}}$$

where y is the observed knee abduction moment, $\hat{y}$ is the predicted moment and N is the number of observations in the test dataset.

The MAE was computed using:

$$MAE=\frac{\sum_{i=1}^{N}y\|\left(i\right)-\hat{y}\left(i\right)\|}{N}$$

The MAPE was computed using:

$$MAPE=\frac{1}{N}\overset{N}{\underset{i=1}{\sum }}\|\frac{y\left(i\right)-\hat{y}\left(i\right)}{y\left(i\right)}\|$$

And finally, the NRMSE which is the RMSE divided by a measure spread. In this work we divide the RMSE by the difference between min and the max of the knee abduction.

$$NRMSE\;\left(\%\right)=\frac{RMSE}{MAX-MIN}\times 100$$

The maximum and minimum values are reported below in the result section.

## 3 Results

For the 33 included participants (female = 15, male = 18), the mean (standard deviation [SD]) age was 39.42 (17.87) years, height was 1.67 m (0.12 m), and body mass was 67.66 kg (12.44 kg). The mean knee abduction moment was −28.06 Nm.s/kg, standard deviation was 11.55 Nm.s/kg, the interquartile range was 15.17 Nm.s/kg, with a variation range (max -min) of 86.94 Nm.s/kg (maximum −88.14 Nm.s/kg, minimum −1.20 Nm.s/kg). The mean (SD) waveforms of our 126 predictors normalized 100% timepoints of the stance phase, on our dataset can be found in the Supplementary Material S1.

Table 2 shows the performance of the five ML models. MAPE and MAE are measures of how far the model’s predictions are off from observed values on average. The baseline model achieved 10.80% with the predicted value spreading on average 2.78 Nm.s/kg from the observed values. Transfer learning with inceptionTime was the best performing model, achieving the best MAPE of 8.28%, which translates to the predicted value spreading on average 1.70 Nm.s/kg from the observed values. In contrast, training the inceptionTime model from scratch resulted in a slightly lower performance compared to transfer learning, with a MAPE of 8.61%. The GADF-xResnet 18 model performed worse than the baseline model with a MAPE of 16.17%. This means that converting a time-series to images did not improve ML prediction performances.

**TABLE 2:** Regression models performance.

	Training set loss (Nm.s/kg)	Validation set loss (Nm.s/kg)	Test set MAE (Nm.s/kg)	Test set RMSE (Nm.s/kg)	Test set MAPE (%)	Test set NRMSE (%)
Baseline model	8.91	16.97	2.78	3.46	10.80	3.98
InceptionTime	6.70	6.05	1.76	2.46	8.61	2.83
Transfer learning	6.54	5.59	1.70	2.36	8.28	2.71
TS-ResNet	5.28	6.10	1.77	2.47	8.65	3.15
GADF-xResnet	19.89	21.33	3.45	4.62	16.17	5.31

## 4 Discussion

The ability to quantify joint moments in the field may revolutionize the clinical management of musculoskeletal disorders where tissue loading has been implicated as a risk factor for the onset, exacerbation, and symptomatic relapse. In partial support of our hypothesis, transfer learning resulted in the best prediction performance of the outcome of knee abduction impulse during walking. However, in contrast to our hypothesis, the GADF-xResnet model was the worst-performing algorithm.

The only other study to our knowledge that investigated the accuracy of ML in predicting KAM impulse was Stetter et al. (2020), which reported an average observed value of 69.16 Nm.s/kg, and a predicted value of 64.23 Nm.s/kg—a difference of 4.93 Nm.s/kg. Given that performance metrics (RMSE, MAE) were not reported for KAM impulse (Stetter et al., 2020), we used the difference in average values as the performance metric for comparison. The performance in predicting KAM impulse in the previous study (Stetter et al., 2020) was worse than all our models tested in the present study. The worse performance by Stetter et al. (2020) could be due to two reasons. First, the previous study used IMU time-series predictors (Stetter et al., 2020) which may be noisier than our kinematic predictors. Second, Stetter et al. (2020) performed validation whereby the training and testing data independent (i.e., subject data in training set not in testing set). However, our training and testing data were dependent, the reason for which was explained in the methods section. Third, they used a fully connected layered neural network model which may not adequately harness the temporal information within the variables (Stetter et al., 2020). As previously mentioned, Boswell et al. (2021) reported that a fully connected network was superior to LTSM network, but the poorer performance of the latter could be an insufficient number of layers. Future investigations are needed to benchmark different types of network architectures on different biomechanical datasets to determine when different modeling approaches would be superior.

Converting the time-series kinematic predictors to images *via* the GADF resulted in the worst prediction performance. This contrasted with another study that reported an improvement in activity classification accuracy from 94% using time-series predictors, to 97% when converting time-series into images as predictors (Boukhennoufa et al., 2021a). The GADF transformed the time-series predictor (300 time points) into 2D images of dimensions 300 × 300 pixels. It is likely that the greater input dimension of the transformed images would require a model with more layers to learn an increased number of parameters, compared to using the original time-series. Another possible reason is that transforming the time series data into images is more fructuous in classification models as opposed to regression models. The ResNet model used in the present study had 18 layers, whilst a previous study used the VGG16 model which had 16 layers (Boukhennoufa et al., 2021a). However, Boukhennoufa et al. (2021a), had only 6 time-series predictors, compared to 126 time-series predictors in the present study. This means that the combination of a high number of predictors coupled with a greater input dimension size means that the number of layers used in our ResNet model was potentially insufficient to learn the parameters.

Another finding of the present study was that our baseline CNN model performed worse than InceptionTime and TS-ResNet, using the same time-series predictors. Both InceptionTime and TS-ResNet contain shortcut residual connections between convolutional layers, whilst our baseline CNN model does not. The benefit of having residual connections within the network is that makes training a deep neural network much easier by reducing the vanishing gradient effect (He et al., 2016). In addition, the high performance of InceptionTime may be attributed to having multiple parallel convolutional layers, each with different filter lengths, learning different latent hierarchical features of the time-series. The benefit of having multiple parallel layers may be analogous to the benefit of ensemble machine learning techniques like boosting—combining the results of multiple weak learners. InceptionTime, when compared to the baseline model, combines multiple extracting structures with different window sizes, which allows the former to extract a more diverse set of features from the predictors than the latter, thereby improving the prediction performance using InceptionTime. In a consistent manner, TS-ResNet’s deep architecture also allows to learn a plethora of features that are associated with this dataset. In contrast, the baseline model likely did not allow to learn the features as well as with InceptionTime and TS-ResNet due to its shallower architecture.

Interestingly, our finding that transfer learning resulted in the best prediction performance was not supported by another study, albeit conducted in running (Liew et al., 2021). In a previous study, the multivariate time-series kinematic predictors were transformed into static images using cubic spline interpolation (Liew et al., 2021). The purpose of the interpolation was so that the predictor dimension fitted the input dimensions of the VGG16 image model used for transfer learning (Liew et al., 2021). For example, from an original array of 490 [observations] × 101 [gait cycle] × 9 [variables] × 3 [axes], the data was transformed into a 490 × 150 × 150 × 3 shape (Liew et al., 2021). This pre-processing step could have introduced excessive noise into the predictors, thereby affecting the prediction performance of transfer learning.

This study has the following limitations. First, we did not perform hyperparameter tuning, and the selected hyperparameters were selected based on the experience of the authors. Hence, our findings can be said to provide a more conservative estimate of the predictive performance of deep and transfer learning models. Second, we used predictors derived from optoelectronic systems, which can still be time-consuming to use in the clinics. Wearable sensors or markerless motion capture represent the most clinically feasible methods of measuring body motions. Whether the performance of the ML approach using these newer technologies would match that of traditional optoelectronic systems needs to be investigated. Third, our model was developed using data collected from treadmill walking, and the performance may be different in overground walking. Lastly, our models were trained to predict a specific load metric, the internal knee abduction impulse. Whether the present study’s findings would similarly translate to other knee load metrics (e.g., peaks), or indeed the entire time-series curve, will need to be investigated.

## 5 Conclusion

We used different state-of-the-art deep learning algorithms to predict knee abduction moment impulse in healthy individuals walking. We found that time-series based deep learning models were superior to an image-based method when predicting knee abduction moment impulse during walking. Also, transfer learning improved the predictive model performance even though the two models are derived from different domain disciplines. Our results support the viability of combining ML with kinematic inputs to quantify biomechanical kinetic measures outside the laboratory. Future studies looking to develop wearable technologies will benefit from knowing the optimal network architecture, and the benefit of transfer learning for predicting joint moments.

## Data Availability

The data and code for this study can be found on the lead author’s GitHub page (https://github.com/el3oss19/dataset).

## Supplementary Material

The Supplementary Material for this article can be found online at: https://www.frontiersin.org/articles/10.3389/fbioe.2022.877347/full#supplementary-material

Supplementary Figure S1 Time-normalized stance-phased plots of the kinematic predictors used for deep learning. Abbreviations: PL—pelvis; TH—thigh; SK—shank; FT—foot; R—right limb; L—left limb; Lin—linear; Ang—angular; displ—displacement; vel—velocity; accl—acceleration; x—anterior-posterior axis; y—vertical axis; z—medial-lateral axis. Units: angular displacement (°), angular velocity (°/s), angular acceleration (°/s^2^), linear displacement (m), linear velocity (m/s), linear acceleration (m/s^2^).
