# Supplementary figures and images for: Predicting the Internal Knee Abduction Impulse During Walking Using Deep Learning
Source: Front Bioeng Biotechnol. 2022 May 12;10:877347. doi: 10.3389/fbioe.2022.877347 (PMC9133596; doi:10.3389/fbioe.2022.877347)

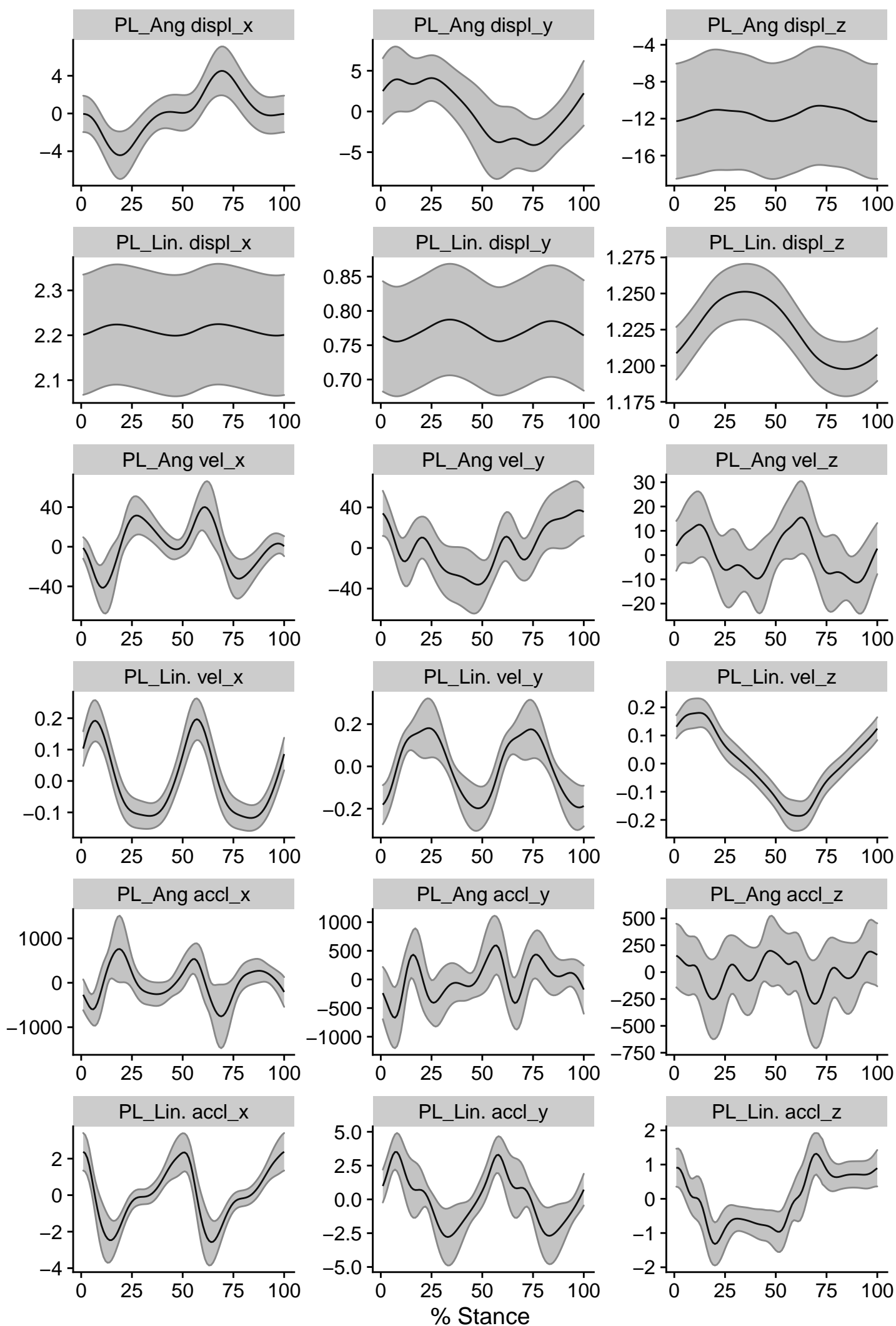

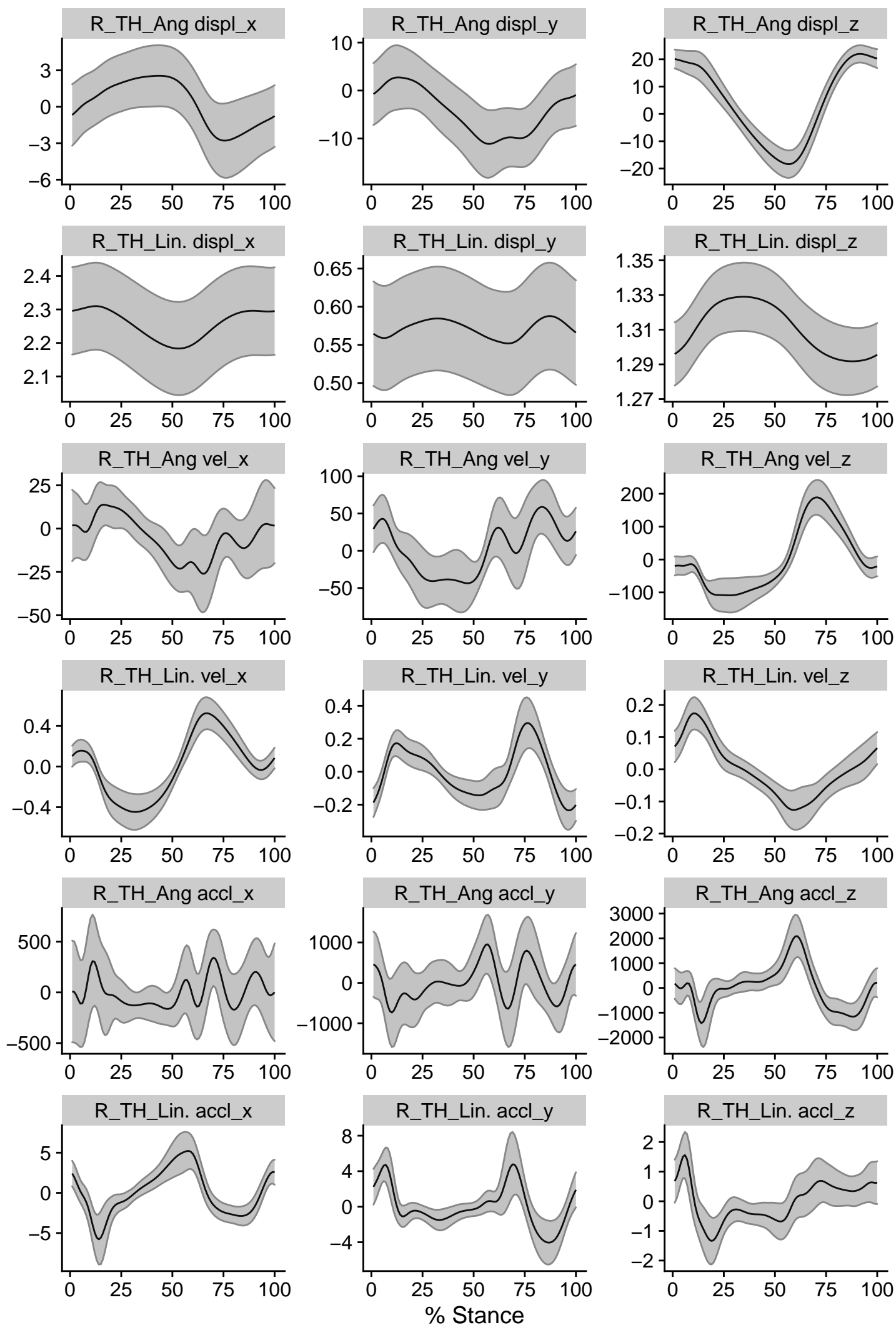

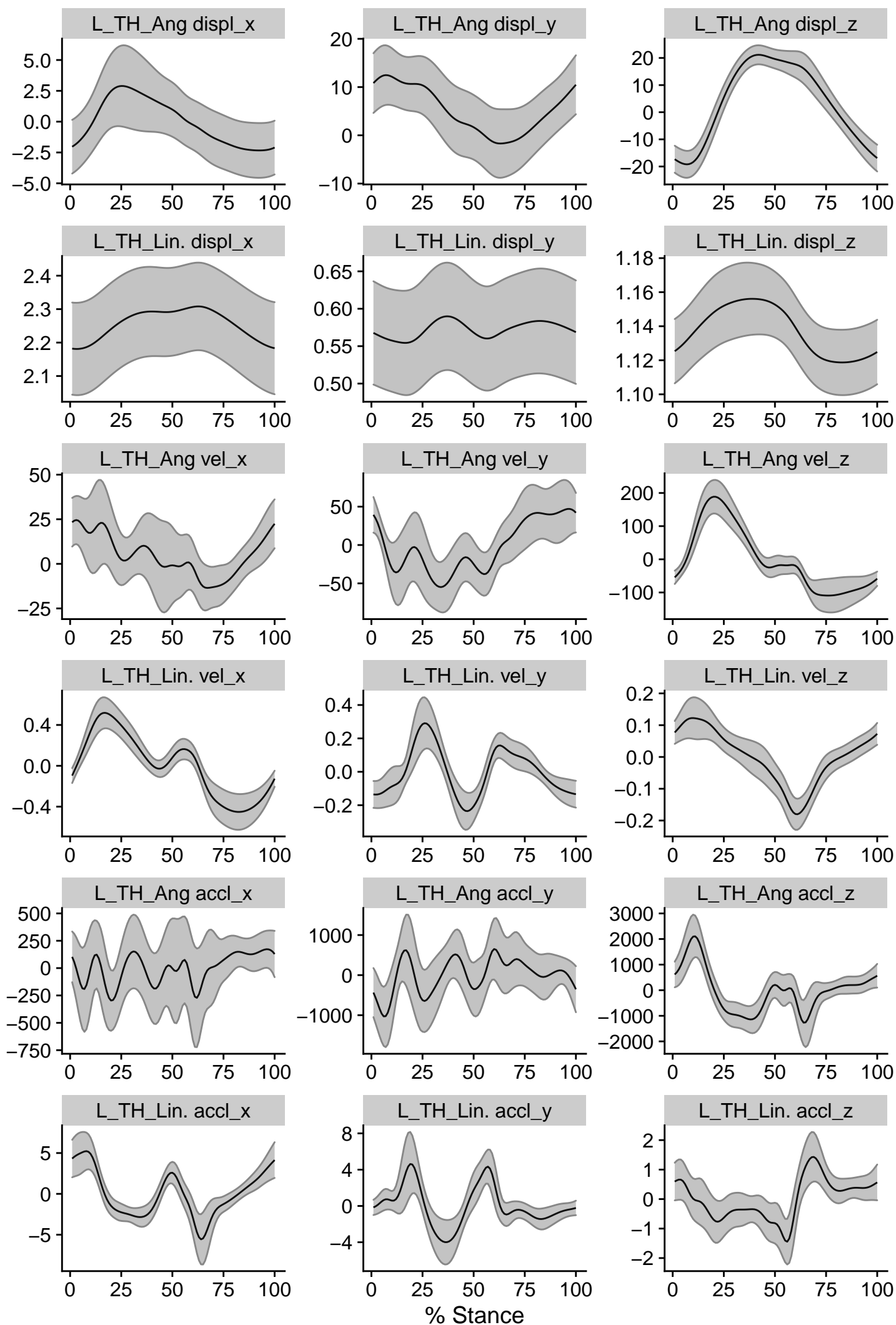

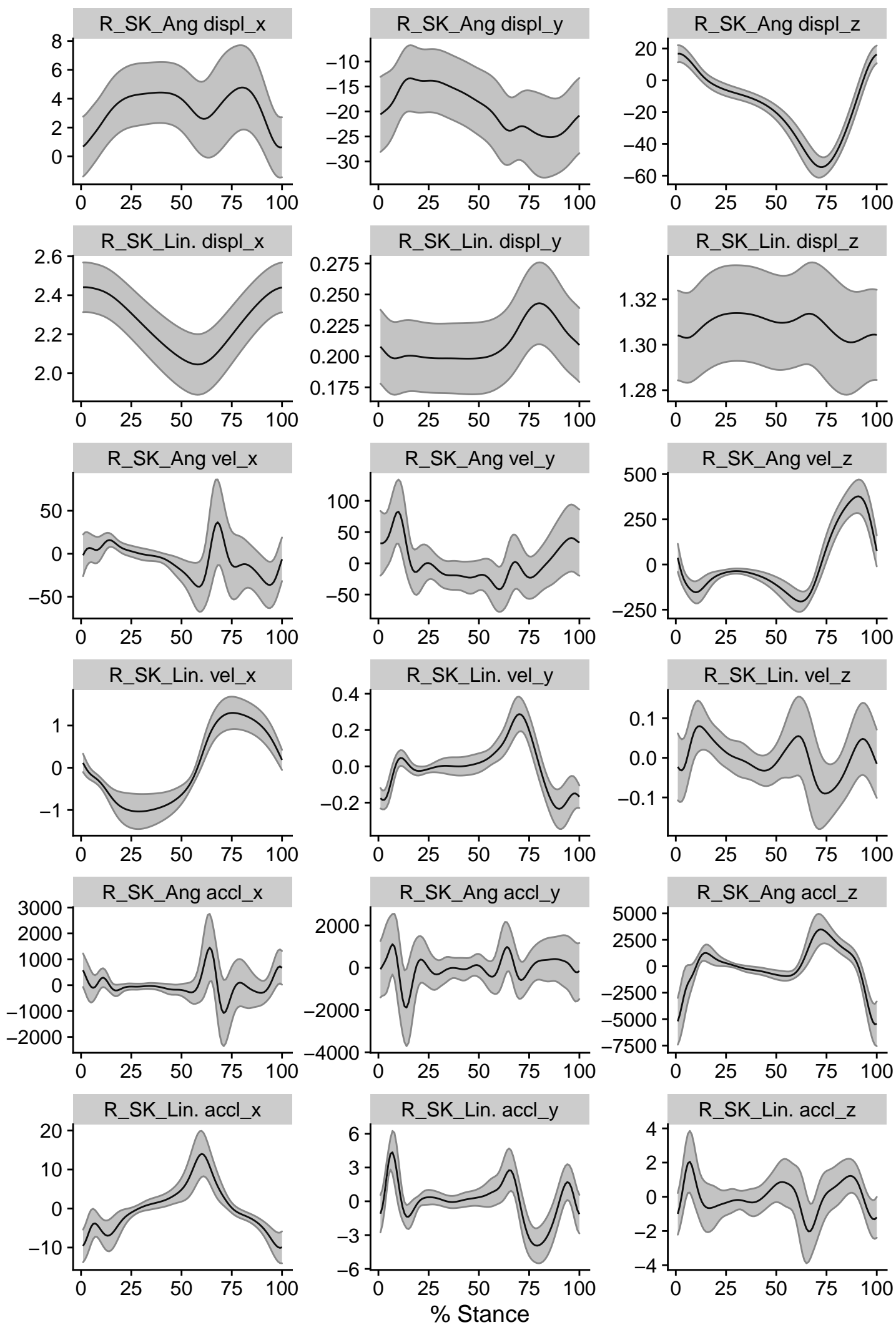

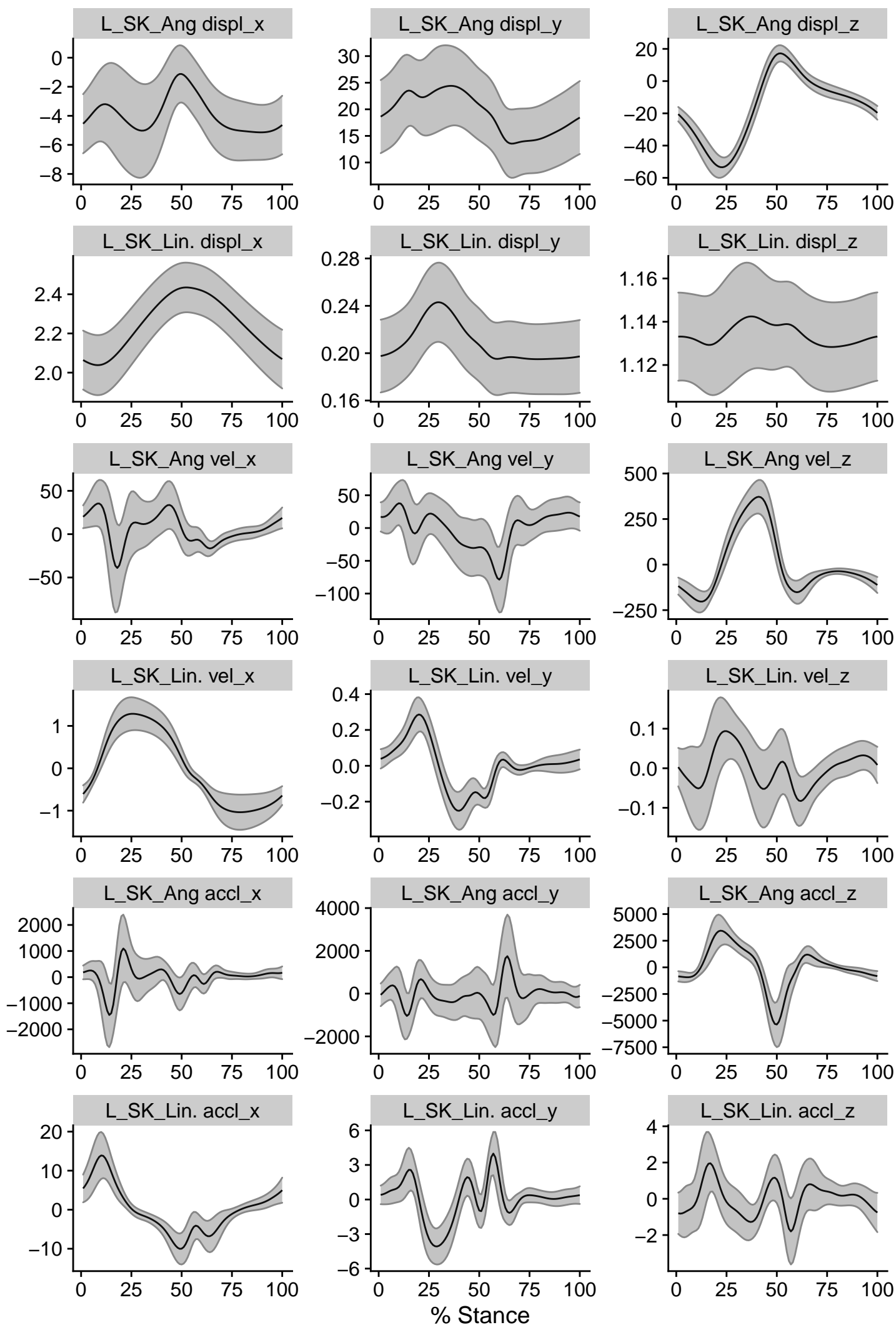

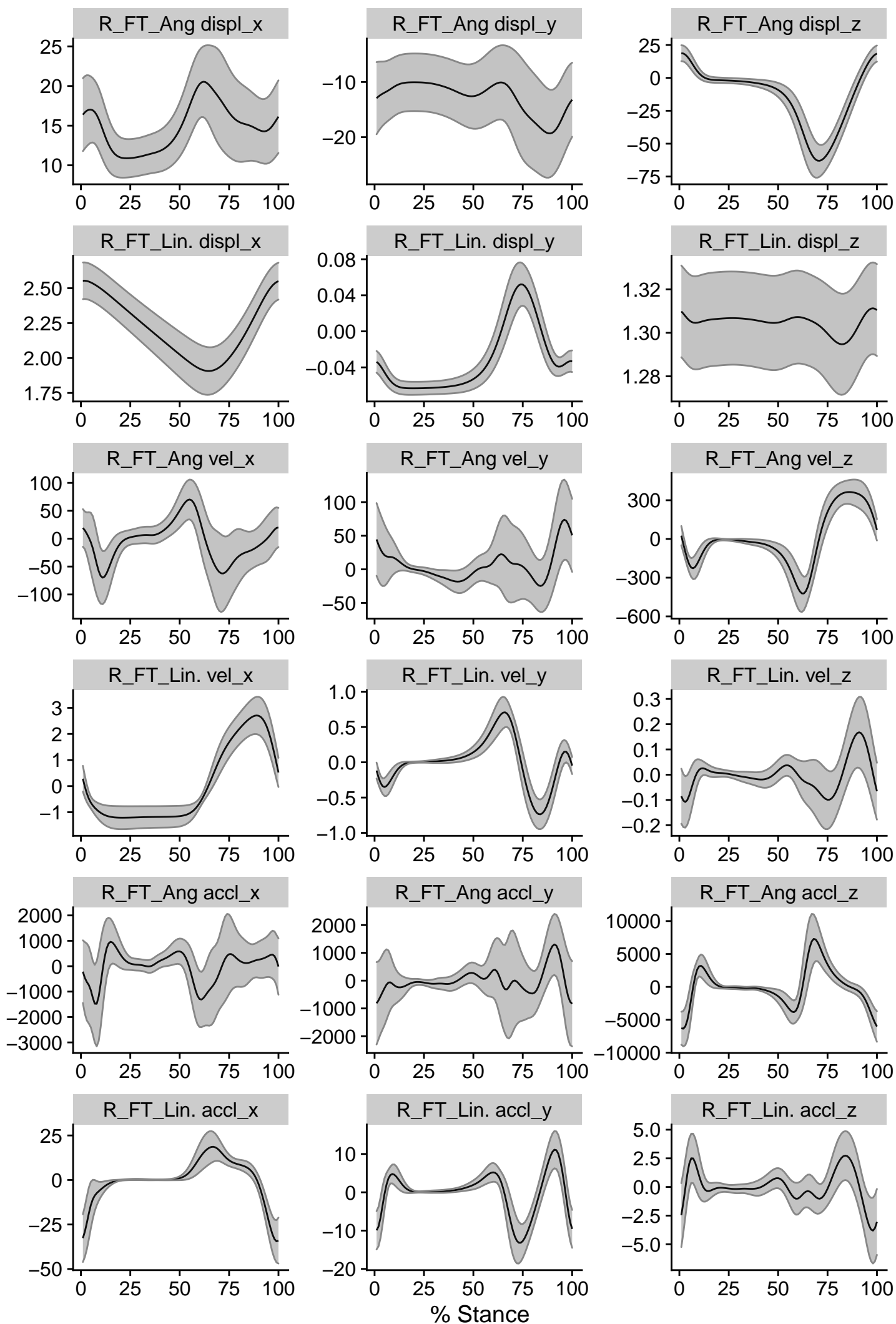

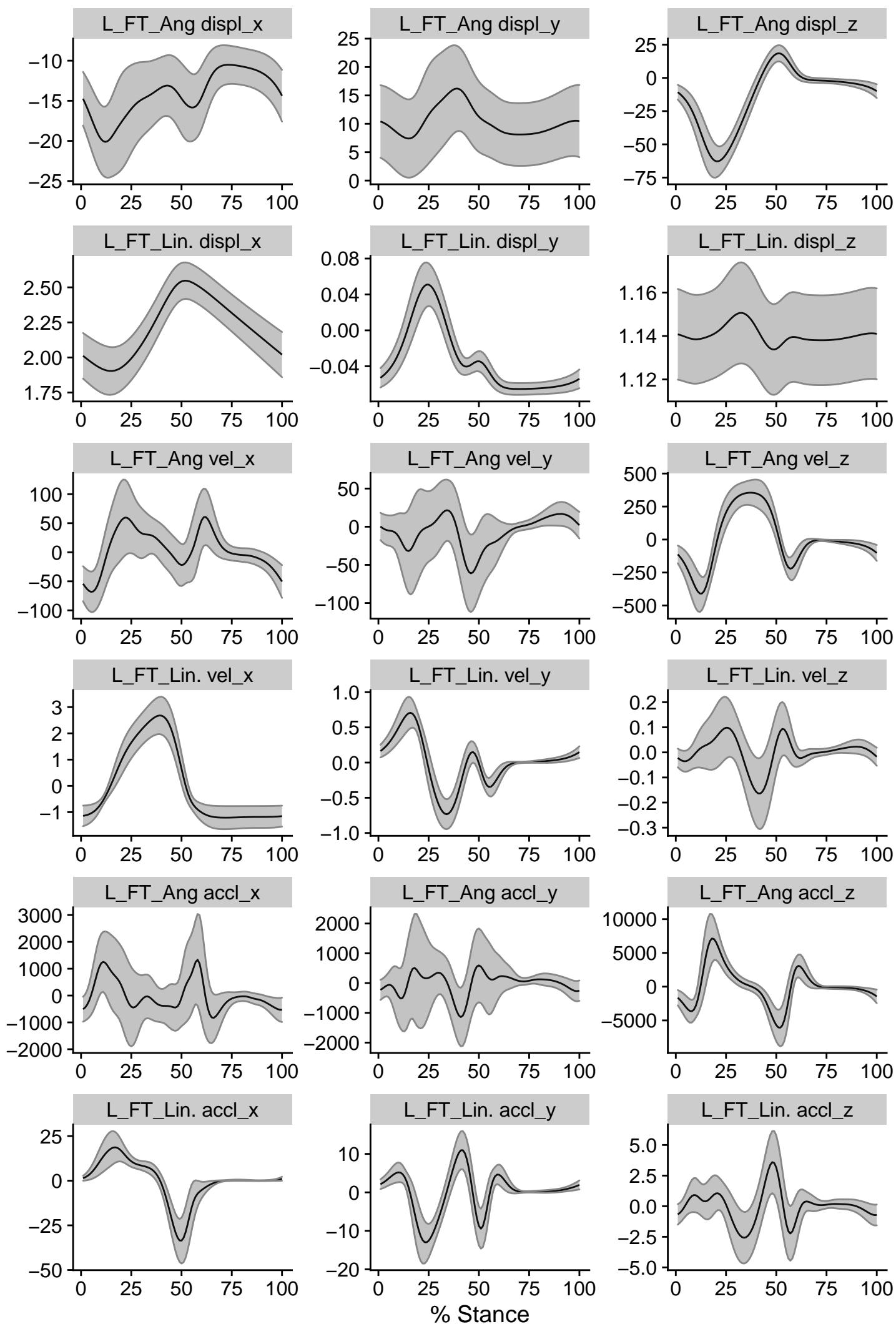

Supplement: Supplementary file 1 [file Datasheet1.PDF]
